# The order of vasopressor discontinuation and incidence of hypotension: a retrospective cohort analysis

**DOI:** 10.1038/s41598-021-96322-7

**Published:** 2021-08-17

**Authors:** Xuan Song, Xinyan Liu, Kimberly D. Evans, Ryan D. Frank, Erin F. Barreto, Yue Dong, Chang Liu, Xiaolan Gao, Chunting Wang, Kianoush B. Kashani

**Affiliations:** 1 Division of Pulmonary and Critical Care Medicine, Department of Medicine, Mayo Clinic, Rochester, MN 55902 USA; 2 ICU, Shandong First Medical University, Shandong, 250117 Shandong China; 3 ICU, Shandong Provincial Hospital Affiliated to Shandong First Medical University, Shandong, 250021 Shandong China; 4 Department of Anesthesiology and Perioperative Medicine, Mayo Clinic, Rochester, MN USA; 5 Department of Biostatistics, Mayo Clinic, Rochester, MN USA; 6 Pharmacy Services, Mayo Clinic, Rochester, MN USA; 7 Robert D. and Patricia E. Kern Center for Science of Health Care Delivery, Rochester, USA; 8 Department of Critical Care Medicine, Zhongnan Hospital of Wuhan University, Wuhan, Hubei China; 9 Department of Critical Care Medicine, Division of Life Sciences and Medicine, The First Affiliated Hospital of USTC, University of Science and Technology of China, Hefei, 230001 Anhui China; 10 Division of Life Sciences and Medicine, University of Science and Technology of China, Hefei, 230001 Anhui China; 11 ICU, Shandong Provincial Hospital Affiliated to Shandong First Medical University, Shandong, 250021 China; 12 Division of Nephrology and Hypertension, Department of Medicine, Mayo Clinic, 200 First Street SW, Rochester, MN 55905 USA

**Keywords:** Diseases, Medical research

## Abstract

The optimal order of vasopressor discontinuation during shock resolution remains unclear. We evaluated the incidence of hypotension in patients receiving concomitant vasopressin (VP) and norepinephrine (NE) based on the order of their discontinuation. In this retrospective cohort study, consecutive patients receiving concomitant VP and NE infusions for shock admitted to intensive care units were evaluated. The primary outcome was hypotension incidence following discontinuation of VP or NE (VP1 and NE1 groups, respectively). Secondary outcomes included the incidence of acute kidney injury (AKI) and arrhythmias. Subgroup analysis was conducted by examining outcomes based on the type of shock. Of the 2,035 included patients, 952 (46.8%) were VP1 and 1,083 (53.2%) were NE1. VP1 had a higher incidence of hypotension than NE1 (42.1% vs. 14.2%; *P* < 0.001), longer time to shock reversal (median: 2.5 vs. 2.2 days; *P* = .009), higher hospital [29% (278/952) vs. 24% (258/1083); *P* = .006], and 28-day mortality [37% (348/952) vs. 29% (317/1,083); *P* < 0.001] when compared with the NE1 group. There were no differences in ICU mortality, ICU and hospital length of stay, new-onset arrhythmia, or AKI incidence between the two groups. In subgroup analyses based on different types of shock, similar outcomes were observed. After adjustments, hypotension in the following 24 h and 28-day mortality were significantly higher in VP1 (Odds ratios (OR) 4.08(3.28, 5.07); *p*-value < .001 and 1.27(1.04, 1.55); *p*-value < .001, respectively). Besides, in a multivariable model, the need for renal replacement therapy (OR 1.68 (1.34, 2.12); *p*-value < .001) was significantly higher in VP1. Among patients with shock who received concomitant VP and NE, the VP1 group was associated with a higher incidence of hypotension in comparison with NE1. Future studies need to validate our findings and their impact on clinical outcomes.

## Introduction

Shock is a life-threatening condition associated with a high rate of mortality^1,2^. Shock state could be due to multiple mechanisms, including decreased cardiac output (cardiogenic), vasodilation (distributive), cardiovascular system obstruction, or reduced effective blood volume (hypovolemic)^3^. One of the primary interventions in the resuscitation of patients with shock states is using a combination of fluids (except in cardiogenic shock) and vasopressors to maintain perfusion to vital organs^4–6^. Vasopressin (VP) and norepinephrine (NE) are the most commonly used vasopressors for the management of shock^7,8^. In catecholamine-resistant patients, high doses of NE alone often fail to reverse shock. Thus, VP may be added to either raise the mean arterial pressure (MAP) or decrease the NE dosage^9^.

When hemodynamic status stabilizes during the shock resolution phase, vasopressor support is gradually tapered to avoid their adverse effects^10^. The 2016 Surviving Sepsis Campaign Guidelines recommended NE as the vasoactive agent of choice with VP as a second-line adjunct^9^. Meanwhile, limited evidence for vasopressor management during the deescalation phase of shock has led to controversies in the field. Bauer et al.^11^ suggest that VP discontinuation before NE may lead to a higher incidence of hypotension in patients recovering from septic shock while receiving concomitant VP and NE, despite the longer half-life of VP (i.e., 1–2 min for NE and 10–35 min for VP). Yet, a similar study by Sacha et al.^12^ observed no significant differences in hypotension incidence based on the order of vasopressors discontinuation.

In this retrospective cohort study, we hypothesize that discontinuing VP before NE is associated with an increased incidence of hypotension, acute kidney injury (AKI), and arrhythmia in patients with shock.

## Materials and methods

This retrospective cohort study was approved by the Mayo Clinic Institutional Review Board (IRB) to use existing medical records among patients who had provided research authorization (approval number: 19–008,234). The need for informed consent was waived by Mayo Clinic IRB due to the minimal risk nature of the study. All methods were performed in accordance with the relevant guidelines and regulations.

### Patients

Patients were eligible for study inclusion if they were, (1) at least 18 years old, (2) admitted to the ICU in Mayo Clinic, Rochester, MN from November 1st, 2007, through January 31st, 2018, (3) diagnosed with shock^13^, and (4) received concomitant NE and VP infusions for ≥ 1 day. We excluded patients who died within 48 h of ICU admission, were terminally ill, had VP and NE discontinued at the same time, or received additional catecholamines and/or inotropes after discontinuation of the first vasopressor. Other vasopressors were allowed if administered before the discontinuation of VP or NE. The order of vasopressor discontinuation was at the discretion of the primary intensivist.

### Data collection

Data collected from electronic medical records included demographics, admitting ICU service, Charlson Comorbidity Index, type of shock (i.e., septic, cardiogenic, or hypovolemic), and hospital and ICU length of stay. Mortality was documented at ICU discharge, hospital discharge, and 28 days. Sequential Organ Failure Assessment (SOFA)^14^ score and mean arterial pressure (MAP) were collected at the time of ICU admission and the time of VP and NE initiation and discontinuation, shock start time, and time and extent of hypotension following discontinuation of the first vasopressor. Maximum VP and NE doses during shock, utilization of renal replacement therapies, and corticosteroids were collected. Besides, new-onset arrhythmias after discontinuation of the first vasopressor, daily serum creatinine, and urine output were documented. To assess study outcomes, treatment for hypotension after discontinuing the first vasopressor (i.e., need for fluid challenge, need for the resumption of the discontinued vasopressor, or need for increased vasopressor dose) was collected.

### Study outcomes

The primary outcome was the incidence of hypotension within 24 h of discontinuation of the first vasopressor. Secondary outcomes included ICU, hospital, and 28-day mortality, ICU and hospital length of stay, ICU readmission, time to shock reversal, new-onset arrhythmias (i.e., ventricular or atrial fibrillation, and/or heart rate > 130), and incidence of AKI.

### Definitions

The shock was defined as MAP < 65 mmHg and serum lactate level > 2 mmol/L. Subgroups were further clarified based on the type of shock. Septic shock was defined based on the sepsis-3 definition^15^. Cardiogenic shock was defined as a state of ineffective cardiac output caused by a primary cardiac disorder that resulted in both clinical and biochemical manifestations of inadequate tissue perfusion^16^. The hypovolemic shock was defined as a condition of tissue hypoperfusion—caused by decreased effective blood volume due to vomiting or diarrhea, severe environmental fluid loss, or rapid and massive blood loss—that led to a decline in aerobic metabolism^17^. Lactate was used to assess hypoperfusion in the patients that clinicians felt were probably in the shock state. Ordering lactic acid for managing shock patients in Mayo Clinic is protocolized. However, physicians can bypass the order when they believe patients are not dealing with a hypoperfusion state. Therefore, all missing lactate levels were imputed as < 2 mmol/L as the clinicians likely felt hypoperfusion was not an issue.

Shock start time (T) is determined as the first instance of MAP < 65 mmHg or serum lactate level > 2 mmol/L, whichever came first. Time zero (T_0_) is the time that the first vasopressor was discontinued. T_1_ is days from T_0_ to the first documented hypotension episode, discharge from ICU, or death. Shock reversal time (T_2_) indicated when MAP of ≥ 65 mmHg was achieved following discontinuation of all vasopressors. The time to shock reversal was calculated as the duration between T_2_ and T.

Hypotension was defined as MAP < 65 mmHg during the first 24 h of discontinuation of the first vasopressor requiring one or more of these interventions: (1) increase in the vasopressor dose, (2) resuming therapy with the discontinued vasopressor or other vasopressors, (3) receipt of at least 500 mL of a crystalloid bolus or 25 g of albumin.

Following the first vasopressor discontinuation time to ICU discharge, all new-onset arrhythmias (i.e., ventricular fibrillation, atrial fibrillation, and/or heart rate > 130 beats per minute) were recorded. AKI was defined based on the Kidney Disease: Improving Global Outcomes (KDIGO) Clinical Practice Guidelines^18^ and monitored from ICU admission up to seven days after the end of vasopressors or hospital discharge, whichever came first.

### Statistical analysis

Baseline demographics and clinical characteristics were summarized using counts and percentages for categorical variables and medians and interquartile ranges for continuous variables. Data distributions based on the first withheld vasopressor, VP or NE, were compared using chi-square and Fisher exact tests^19^ (where appropriate) for categorical variables and Wilcoxon rank-sum tests for continuous variables. Chi-square tests and Wilcoxon rank-sum tests were used to determine associations between outcomes and the first withheld vasopressor.

Cumulative incidence curves of hypotension were plotted for visualization purposes. These same comparisons were made for septic-, hypovolemic-, and cardiogenic-only shock patients. SAS version 9.4 (SAS Institute Inc., Cary, NC) was used for all analyses. A two-sided *p*-value < 0.05 was considered statistically significant.

### Ethical approval

This retrospective cohort study was approved by the Mayo Clinic Institutional Review Board (IRB) to use existing medical records among patients who had provided research authorization (Approval No.: 19-008234).

### Consent to participate

The need for informed consent was waived due to the minimal risk nature of the study.

## Results

### All shock patients

**Figure 1:**
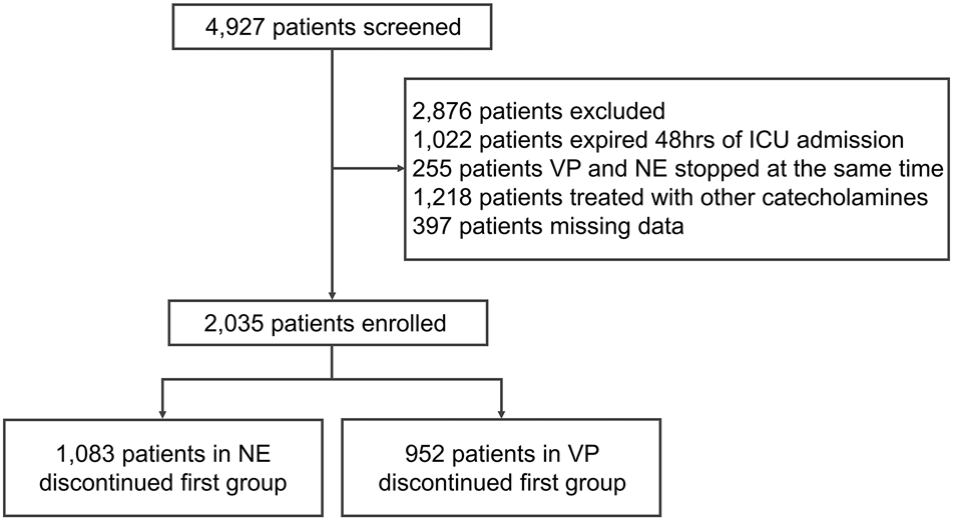
Study flow chart of patient enrollment.

We initially screened 4,927 patients, of which 2,035 met all inclusion criteria after 2,876 were excluded (Fig. 1). Of those included, 952 (47%) were in the VP-discontinued-first (VP1) group, and 1,083 (53%) were in the NE-discontinued-first (NE1) group. Demographics and patient characteristics for each group are presented in Table 1. The groups had similar demographic characteristics and severity of illness (SOFA score, median 10 vs. 10; *P* = 0.3). VP1 patients had higher Charlson Comorbidity Index [median 5 (4,8) vs. 5 (3,7); *P* < 0.001], incidence of cancer [36% (343/952) vs. 30% (322/1,083); *P* = 0.003], sepsis [55% (521/952) vs. 41% (440/1083)], diabetes mellitus [28% (267/952) vs. 23% (251/1,083); *P* = 0.01], corticosteroid use [50% (473/952) vs. 33% (357/1083); *P* < 0.001], dialysis requirement [24% (227/952) vs. 16% (170/1,083); *P* < 0.001], NE dose, and lower MAP when compared to NE1 patients.

**Table 1:** Demographics and patient characteristics of all shock patients.

	NE1 N = 1,083	VP1 N = 952	*P*-value
Age, yr	65 (54, 74)	66 (56, 75)	.053 ^†^
Male sex	672 (62%)	574 (60%)	.4 ^‡^
Weight, kg, n = 1936	83 (69, 100)	82 (68, 98)	.4 ^†^
White race	975 (90%)	850 (89%)	.6 ^‡^
SOFA score, n = 2034	10 (8, 12)	10 (8, 12)	.3 ^†^
Charlson score	5 (3, 7)	5 (4, 8)	< .001 ^†^
**Comorbid disease**			
Heart disease	268 (25%)	209 (22%)	.1 ^‡^
Pulmonary disease	194 (18%)	180 (19%)	.6 ^‡^
Immunodeficiency	38 (4%)	29 (3%)	.6 ^‡^
Liver disease	42 (4%)	40 (4%)	.7 ^‡^
Kidney disease	237 (22%)	191 (20%)	.3 ^‡^
Diabetes mellitus	251 (23%)	267 (28%)	.01 ^‡^
Cancer tumor	322 (30%)	343 (36%)	.003 ^‡^
Other	193 (18%)	195 (21%)	.1 ^‡^
**Shock type**			< .001 ^‡^
Septic only	440 (41%)	521 (55%)	
Hypovolemic only	16 (2%)	16 (2%)	
Cardiogenic only	164 (15%)	93 (10%)	
Two types	113 (10%)	94 (10%)	
Three types	8 (1%)	9 (1%)	
Unknown	342 (32%)	219 (23%)	
Corticosteroid	357 (33%)	473 (50%)	< .001 ^‡^
Requirement for dialysis	170 (16%)	227 (24%)	< .001 ^‡^
Maximum NE dose; µg/kg/min	0.15 (0.08, 0.30)	0.21 (0.12, 0.40)	< .001 ^†^
Maximum VP dose; µg/kg/min	0.04 (0.04, 0.04)	0.04 (0.04, 0.04)	< .001 ^†^
NE end dose, n = 2,034	0.02 (0.01, 0.03)	0.02 (0.01, 0.03)	< .001 ^†^
VP end dose, n = 2,022	0.03 (0.02, 0.04)	0.04 (0.02, 0.04)	< .001 ^†^
VP within 3 h from shock start	395 (37%)	370 (39%)	.3 ^‡^
MAP at first vasopressor initiation; mmHg	68 (60, 77)	66 (60, 76)	.1 ^†^
MAP at first vasopressor discontinuation; mmHg	75 (68, 84)	74 (67, 82)	.02 ^†^
**Interventions after hypotension**			
Crystalloids > 500 ml;	19 (2%)	23 (2%)	.3 ^‡^
Albumin > 25 g	11 (1%)	11 (1%)	.8 ^‡^
VP restart	120 (11%)	0 (0.0%)	< .001 ^‡^
NE restart	5 (1%)	385 (40%)	< .001 ^‡^
VP increase dose	55 (5%)	0 (0.0%)	< .001 ^‡^
NE increase dose	3 (0.3%)	287 (30%)	< .001 ^§^

Numbers indicate N (%) and (minimum, maximum) unless otherwise noted.

^†^Wilcoxon rank-sum; ^‡^Chi-square; ^§^Fisher exact.

Table 2 shows the outcomes of the cohort. The VP1 group had higher incidence of hypotension [42% (401/952) vs. 14% (154/1,083); *P* < 0.001], longer time to shock reversal (median: 3[2, 5] vs. 2[1, 4] days; *P* = 0.009), higher hospital [29% (278/952) vs. 24% (258/1,083); *P* = 0.006], and 28-day mortality [37% (348/952) vs. 29% (317/1,083); *P* < 0.001] when compared with the NE1 group. There were no differences in ICU mortality, ICU, hospital length of stay, new-onset arrhythmia, or AKI incidence.

**Table 2:** Clinical outcomes of all shock patients.

	NE1 N = 1,083	VP1 N = 952	*p*-value
Incidence of hypotension within 24 h of first vasopressor stopped	154 (14%)	401 (42%)	< .001 †
ICU mortality	177 (16%)	180 (19%)	.1 †
Hospital mortality	258 (24%)	278 (29%)	.006 †
28-day mortality	317 (29%)	348 (37%)	< .001 †
ICU length of stay, days	6 (4, 12)	6 (4, 12)	.9 ‡
Hospital length of stay, days	16 (9, 31)	16 (9, 31)	.7 ‡
ICU readmission	137 (13%)	139 (15%)	.2 †
Time of shock reversal, days, n = 1670	2.2 (1.4, 4.3)	2.5 (1.5, 5.3)	.009 ‡
Incidence of new-onset arrhythmias	487 (45%)	412 (43%)	.4 †
AKI	608 (56%)	573 (60%)	.1 †
**AKI stage**			.9 †
I	176 (29%)	161 (28%)	
II	207 (34%)	194 (34%)	
III	225 (37%)	218 (38%)	

Numbers indicate N (%) and (minimum, maximum) unless otherwise noted.

^†^Chi-square; ‡Wilcoxon rank-sum.

Following adjustments for age, Charlson comorbidity index, SOFA score, maximum norepinephrine dose, corticosteroid use, diabetes mellitus, sepsis, and need for dialysis, when vasopressin stopped first hypotension in the following 24 h (Odds ratio (OR) 4.08 (3.28, 5.07); *p*-value < 0.001) and 28-day mortality (OR 1.27 (1.04, 1.55); *p*-value < 0.001) was significantly higher when compared with those who stopped norepinephrine first. Besides, after adjustment for age, Charlson comorbidity index, SOFA score, and maximum norepinephrine dose, the need for renal replacement therapy (OR 1.68 (1.34, 2.12); *p*-value < 0.001) was significantly higher when vasopressin stopped before norepinephrine.

### Subgroup: septic shock patients

The septic shock subgroup consisted of 961 patients, with 521 (54%) in the VP1 group and 440 (46%) in the NE1 group. As shown in Additional Table 1, the VP1 group had higher use of corticosteroid [63% (326/521) vs. 47% (207/440); *P* < 0.001], dialysis requirement [23% (121/521) vs. 18% (79/440); *P* < 0.001], and NE max dose [0.24 (0.13, 0.47) vs. 0.28 (0.17, 0.48) µg/kg/min; *P* = 0.004] than the NE1 group. The VP1 group also had higher incidence of hypotension [48% (250/521) vs. 15% (66/440); *P* < 0.001], along with shorter ICU [6 (3, 10) vs. 7 (4, 13); *P* < 0.001] and hospital length of stay [15 (9, 31) vs. 19 (10, 38); *P* = 0.002] as compared to the NE1 group (Additional Table 2).

### Subgroup: hypovolemic shock patients

The hypovolemic shock subgroup consisted of 32 patients (Additional Table 3). VP1 patients had lower weight [74 (60, 92) vs. 100 (84, 129); *P* = 0.02] and higher NE max dose [0.20 (0.13, 0.28) vs. 0.10 (0.05, 0.15) µg/kg/min; *P* = 0.002] compared to NE1 patients. There were no significant differences in outcomes (Additional Table 4).

### Subgroup: cardiogenic shock patients

There were 257 patients in the cardiogenic shock subgroup, of which 93 (36%) were in the VP1 group, and 164 (64%) were in the NE1 group. Demographics and patient characteristics for each group are presented in Additional Table 5. The VP1 group had less heart disease [28 (30%) vs. 70 (43%); *P* = 0.046], more diabetes mellitus [32 (34%) vs. 37 (23%); *P* = 0.04], and higher max NE dose [0.12 (0.09, 0.20) vs. 0.10 (0.06, 0.15) µg/kg/min; *P* = 0.005] than the NE1 group. The VP1 group had a higher incidence of hypotension compared to the NE1 group [24% (22/93) vs. 13% (21/164); *P* = 0.03], but there were no differences in other secondary outcomes (Additional Table 6).

### Cumulative incidence of hypotension and shock reversal

**Figure 2:**
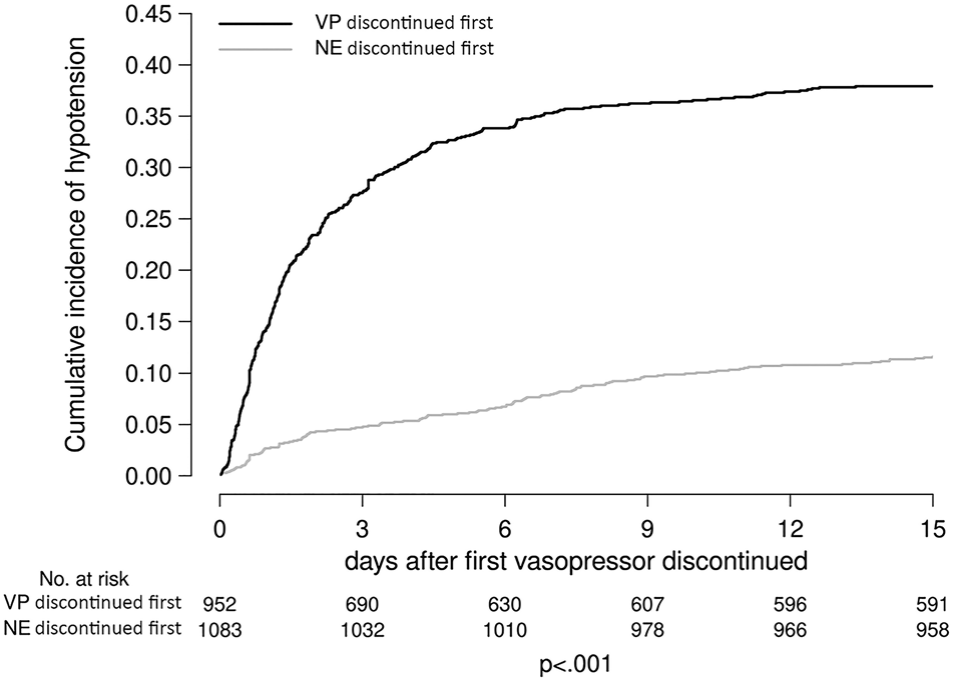
Cumulative incidence of hypotension following first vasopressor discontinuation by order of vasopressor discontinued.

**Figure 3:**
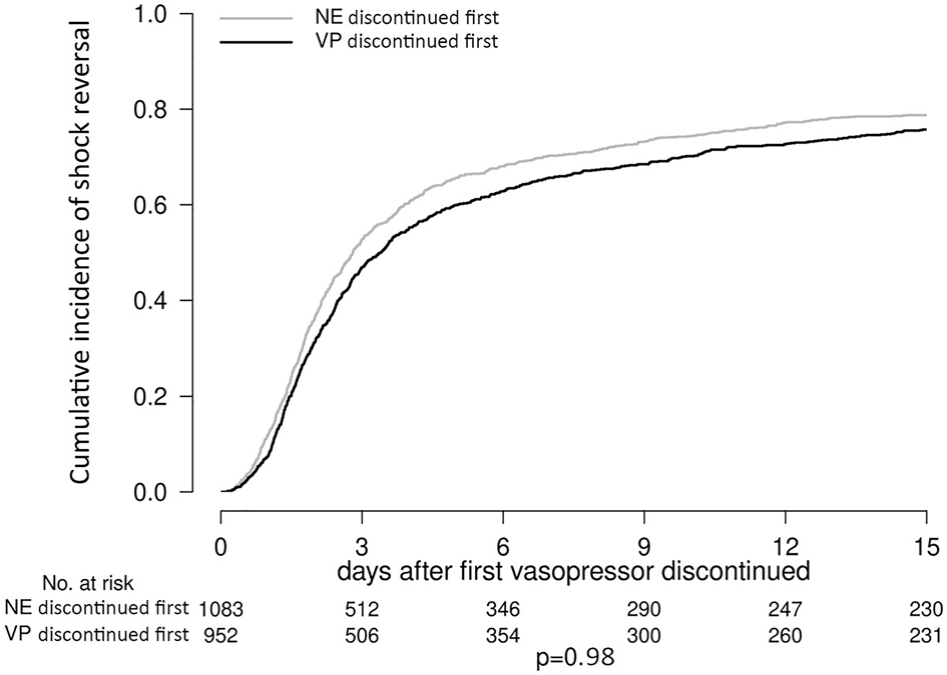
Cumulative incidence of shock reversal following first vasopressor discontinuation by order of vasopressor discontinued.

The cumulative incidence of hypotension following the first vasopressor discontinuation is presented in Fig. 2. By day fifteen, the VP1 group had a significantly higher incidence of hypotension than the NE1 group (37% vs. 11%, *P* < 0.001). The cumulative incidence of shock reversal based on the first discontinued vasopressor is presented in Fig. 3. The NE1 group shock reversal was significantly higher than for VP1 patients (*P* = 0.01), i.e., in the NE1 group, 78.8% had shock reversal by day 15, compared to 75.7% in the VP1 group.

Among septic patients, the VP1 group had a significantly higher incidence of hypotension following the first vasopressor discontinuation relative to the NE1 group (Additional Figure 1, *P* < 0.001), but no difference in the incidence of shock reversal between these groups noted (Additional Figure 2, *P* = 0.4). In hypovolemic shock, between VP1 and NE1 groups, there was no significant difference in the incidence of hypotension (Additional Figure 3, *P* = 0.3) or shock reversal based on vasopressor order discontinuation (Additional Figure 4, *P* = 0.06). Cardiogenic shock patients were similar to septic shock patients in that there were significant differences in the cumulative incidence of hypotension based on the vasopressor discontinuation order (Additional Figure 5, *P* = 0.02). Still, no difference in the rate of shock reversal was seen (Additional Figure 6, *P* = 0.1).

## Discussion

In this retrospective cohort study of ICU patients receiving concomitant VP and NE treatment for shock management, there was a higher incidence of hypotension when VP was discontinued before NE. Additionally, when VP stopped first, a longer time to shock reversal, increased hospital mortality, and increased 28-day mortality were noted when compared with discontinuation of NE first. Order of vasopressor discontinuation did not affect other clinical outcomes, including ICU and hospital length of stay, ICU readmission, ICU mortality, the incidence of new-onset arrhythmia, and AKI. Subgroup analysis of patients with septic and cardiogenic shock revealed similar conclusions, i.e., discontinuing VP before NE was correlated with a higher incidence of hypotension but not associated with other ICU-related clinical outcomes.

The Surviving Sepsis Campaign guidelines for clinical management of septic shock suggest norepinephrine as the first-line vasopressor, and vasopressin could be added in case of catecholamine-resistant hypotension^9^. However, these guidelines do not provide recommendations regarding vasopressor discontinuation order when patients need NE and VP simultaneously, which is likely due to the sparsity of data regarding the benefits or disadvantages of vasopressor deescalation order. Therefore, this choice is left to the clinicians' discretion. Based on the VASST trial results and similar to most clinical practices, VP was not titrated off in our study, and it was turned on and off based on patient needs^20^. Thus, in the VP1 group, VP was discontinued when patients' needs for NE were on a downward trajectory. On the contrary, in the NE1 group, NE was titrated off before VP was turned off.

Only limited data explicitly evaluate the discontinuation order of VP and NE in septic shock patients^11,21,22^. In a retrospective study, Bauer et al.^11^ found that those who had VP stopped first developed hypotension more frequently (56% vs. 16%; *P* = 0.008) and had earlier (median 1.7 vs. 7.3 h, *P* = 0.04) hypotension. In another large, retrospective cohort study, Sacha et al.^12^ evaluated 585 patients in the recovery phase of septic shock. They found no significant difference in hypotension incidence based on the discontinuation order of VP and NE (55% vs. 50%, *P* = 0.3). Our findings are similar to those of Bauer et al. and contribute valuable evidence to the literature by including a larger cohort of septic shock patients. In performing several subgroup analyses, we found the association between VP discontinuation and hypotension to be remarkably consistent, adding to the strength of our conclusions.

The results of this study should be interpreted in the context of varying subgroup sizes and possible confounders. Of note, septic shock was by far the largest subgroup in this study and demonstrated statistically significant differences in the primary outcome of hypotension; the next largest subgroup, i.e., cardiogenic shock, also reached statistically significant results in the primary outcome (though to a slightly lesser extent); and the smallest cohort of hypovolemic shock did not show statistically significant differences in hypotension based on vasopressor discontinuation order. It remains undetermined whether different types of shock patients respond differently to vasopressor discontinuation order or whether more statistical power is needed to elucidate commonalities among them. Thus, interpretation of our findings for the smallest subgroup, i.e., hypovolemic shock, should be made cautiously, and generalizations need to be avoided. These questions merit further investigation.

There may be several biomolecular and physiological advantages of continued VP therapy during shock resolution. VP is a cyclic nano-peptide hormone, also known as the antidiuretic hormone, that plays an important role in the cardiovascular system's homeostatic mechanisms, exhibiting multiple hormonal and osmoregulatory effects beyond its pressor activity^23,24^. The decrease in VP levels increases the possibility of relative VP deficiency as a critical factor in persistent vasodilatory shock^25–27^. VP administration could decrease catecholamine requirement, thus reducing their adverse effects, including arrhythmia, acrocyanosis, vasospasm. Additionally, VP administration could lead to decreased renin-aldosterone-angiotensin system and neurohormonal activation, inhibition of proinflammatory cytokines, improvement in calcium handling, and potentiation of endogenous glucocorticoids^28–30^.

Based on the previous studies indicating VP contribution to the reduction of atrial fibrillation (by sparing adrenergic stimulation provided by catecholaminergic vasopressors)^13,31–33^, we hypothesized a higher rate of arrhythmia among patients who had VP discontinued first. However, the incidence of new-onset arrhythmia was not associated with the order of vasopressor withholding in this study. This may be related to type II error caused by inadequate power in a relatively small sample size.

Likewise, previous investigations showed that sustained VP therapy could be kidney-protective^34–36^, based on VP maintenance of glomerular filtration rate and improved creatinine clearance compared with NE^37,38^. However, we found no such protective effect in our study as there was no significant difference in AKI incidence between VP and NE discontinued first groups. Inadequate sample size, the low sensitivity of AKI definition criteria for acute tubular injury, and a short follow-up period for AKI development could have led to this finding.

It has been proposed that the combination of VP and corticosteroids could result in shorter shock duration and improved survival in patients with sepsis^39,40^. Several possible biological explanations exist for these interactions, including VP binding to V1b receptors in the anterior pituitary, leading to adrenocorticotropin hormone release^41^. On the other hand, corticosteroids restore cytokine-mediated down-regulation of VP receptors^42^. This suggests a regulatory interdependence between VP and cortisol secretion when VP stimulates corticotropin production in the setting of relative adrenal insufficiency^43^. Nevertheless, the interaction of corticosteroids and VP in the physiologic response to and vasodilatory shock management remains controversial. We observed more corticosteroids used in the VP1 patients in all-shock and septic shock cohorts, so further studies are needed to clarify whether corticosteroids influence the higher incidence of hypotension in the discontinued VP first group. Interestingly, in septic shock cohorts, we observed that VP1 patients stayed in the ICU for less time than NE1 patients, which is inconsistent with previous studies. This may be related to more corticosteroids being used in the VP1 group^44,45^, thus introducing bias to the result. Corticosteroids may help in vascular sensitivity to catecholamines through an increase in adrenoceptor gene expression^46^. Vasopressin has also been demonstrated to increase cortisol and adrenocorticotropic hormone, potentially assisting with the relative adrenal insufficiency observed in septic shock^47^.

Our study should be interpreted carefully after considering its limitations. First, this is a single-center, retrospective study and therefore is less generalizable and robust than a multi-site study with randomization of vasopressor discontinuation order. Particularly, knowing that the white race comprised more than 90% of participants, our results need to be validated on other races and groups. While it is physiologically plausible to observe differences in episodes of hypotension based on different strategies in the use of vasopressors, the hard clinical outcomes, including mortality, that were statistically different between the two groups, need validation in prospective studies as we were only able to establish correlations rather than causal relationships. Second, due to a lack of protocol for the discontinuation order, the order of vasopressor discontinuation was at the bedside practitioner's discretion. As such, it could be possible that VP was discontinued before NE because of an adverse drug event or some other medically necessary reason/s rather than simply practitioner preference. This could have induced a bias in our results which need to be validated in prospective studies. A note should also be made that inotropic and cardiac support devices for patients in the cardiogenic shock subgroup were beyond the scope of this study. However, they could likely have played a critical role in patient care and recovery. Finally, this study did not investigate cost-effectiveness. Considering the expenses associated with VP^48^, the cost-effectiveness of continuing VP therapy for longer periods is of paramount interest.

## Conclusion

In septic and cardiogenic shock patients who received concomitant VP and NE, VP discontinuation before NE was associated with a higher incidence of hypotension than with discontinuation of NE before VP. Apart from hospital and 28-day mortality rates, the discontinuation order did not correlate with other major clinical outcomes. We noted similar results in both the septic and cardiogenic shock patients.

## Supplementary Information

**Additional Table 1.** Demographics and Patient Characteristics Among Septic Shock Only.

**Additional Table 2.** Clinical Outcomes of Septic Shock Only.

**Additional Table 3.** Demographics and Patient Characteristics Among Hypovolemic Shock Only.

**Additional Table 4.** Clinical Outcomes of Hypovolemic Shock Only.

**Additional Table 5.** Demographics and Patient Characteristics Among Cardiogenic Shock Only.

**Additional Table 6.** Clinical Outcomes of Cardiogenic Shock Only.

**Additional Figure 1.** Cumulative incidence of hypotension following first vasopressor discontinuation among septic shock only patients.

**Additional Figure 2.** Cumulative incidence of shock reversal following first vasopressor discontinuation among septic shock only patients.

**Additional Figure 3.** Cumulative incidence of hypotension following first vasopressor discontinuation among hypovolemic shock only patients.

**Additional Figure 4.** Cumulative incidence of shock reversal following first vasopressor discontinuation among hypovolemic shock only patients.

**Additional Figure 5.** Cumulative incidence of hypotension following first vasopressor discontinuation among cardiogenic shock only patients.

**Additional Figure 6.** Cumulative incidence of shock reversal following first vasopressor discontinuation among cardiogenic shock only patients.

## Data Availability

The data used for this research are available from the corresponding author on reasonable request and subject to Institutional Review Board guidelines.

## Abbreviations

VP: Vasopressin
NE: Norepinephrine
AKI: Acute kidney injury
VP1: Vasopressin-discontinued-first
NE1: Norepinephrine-discontinued-first
MAP: Mean arterial pressure
SOFA: Sequential Organ Failure Assessment
ICU: Intensive care unit
