# Supplementary material for: The order of vasopressor discontinuation and incidence of hypotension: a retrospective cohort analysis
Source: Sci Rep. 2021 Aug 17;11:16680. doi: 10.1038/s41598-021-96322-7 (PMC8371115; doi:10.1038/s41598-021-96322-7)
Supplement: Supplementary file 1 — Additional Table 1. Demographics and Patient Characteristics Among Septic Shock Only. [file 41598_2021_96322_MOESM1_ESM.docx]

**Additional Table 1.** Demographics and Patient Characteristics Among Septic Shock Only

| Characteristic | NE1 N=440 | VP1 N=521 | p-value |
| --- | --- | --- | --- |
| Age, yr | 66 (56, 76) | 66 (55, 76) | .7 † |
| Male sex | 263 (60%) | 292 (56%) | .2 ‡ |
| Weight, kg, n=911 | 79 (66, 97) | 81 (67, 97) | .5 † |
| White race | 400 (91%) | 467 (90%) | .4 § |
| SOFA score | 10 (8, 13) | 10 (8, 12) | .7 † |
| Charlson score | 6 (3, 8) | 6 (4, 8) | .3 † |
| Comorbid disease |  |  |  |
| Heart disease | 77 (18%) | 107 (21%) | .2 ‡ |
| Pulmonary disease | 87 (20%) | 109 (21%) | .7 ‡ |
| Immunodeficiency | 20 (5%) | 13 (3%) | .1 ‡ |
| Liver disease | 27 (6%) | 21 (4%) | .1 ‡ |
| Kidney disease | 89 (20%) | 105 (20%) | .9 ‡ |
| Diabetes mellitus | 114 (26%) | 146 (28%) | .5 ‡ |
| Cancer tumor | 174 (40%) | 224 (43%) | .3 ‡ |
| Other | 84 (19%) | 106 (20%) | .6 ‡ |
| Corticosteroid | 207 (47%) | 326 (63%) | <.001 ‡ |
| Requirement for dialysis | 79 (18%) | 121 (23%) | .04 ‡ |
| Maximum NE dose; µg/kg/min | 0.24 (0.13, 0.47) | 0.28 (0.17, 0.48) | .004 † |
| Maximum VP dose; µg/kg/min | 0.04 (0.04, 0.04) | 0.04 (0.04, 0.04) | .02 † |
| NE end dose; µg/kg/min | 0.02 (0.01, 0.04) | 0.02 (0.01, 0.03) | .7 † |
| VP end dose; µg/kg/min | 0.04 (0.03, 0.04) | 0.04 (0.03, 0.04) | .1 † |
| VP within 3 hrs from shock start | 137 (31%) | 192 (37%) | .1 ‡ |
| MAP at first vasopressor initiation; mmHg | 66 (58, 73) | 65 (59, 73) | .5 † |
| MAP at first vasopressor discontinuation; mmHg | 74 (67, 82) | 72 (66, 80) | .1 † |
| Interventions after hypotension |  |  |  |
| Crystalloids >500ml | 8 (2%) | 15 (3%) | .3 ‡ |
| Albumin >25g | 7 (2%) | 5 (1%) | .4 ‡ |
| VP restart | 49 (11%) | 0 (0.0%) | <.001 ‡ |
| NE restart | 2 (1%) | 239 (46%) | <.001 § |
| VP increase dose | 13 (3%) | 0 (0.0%) | <.001 ‡ |
| NE increase dose | 1 (0.2%) | 175 (34%) | <.001 § |
| Numbers indicate N (%) and (minimum, maximum) unless otherwise noted. † Wilcoxon rank-sum ‡ Chi-square § Fisher exact | | | |
