# Supplementary material for: The order of vasopressor discontinuation and incidence of hypotension: a retrospective cohort analysis
Source: Sci Rep. 2021 Aug 17;11:16680. doi: 10.1038/s41598-021-96322-7 (PMC8371115; doi:10.1038/s41598-021-96322-7)
Supplement: Supplementary file 2 — Additional Table 2. Clinical Outcomes of Septic Shock Only. [file 41598_2021_96322_MOESM2_ESM.docx]

**Additional Table 2.** Clinical Outcomes of Septic Shock Only

| Characteristic | NE1 N=440 | VP1 N=521 | p-value |
| --- | --- | --- | --- |
| Incidence of hypotension within 24 hours of first vasopressor stopped | 66 (15%) | 250 (48%) | <.001 † |
| ICU mortality | 88 (20%) | 95 (18%) | .5 † |
| Hospital mortality | 135 (31%) | 162 (31%) | .9 † |
| 28-day mortality | 168 (38%) | 206 (40%) | .7 † |
| ICU length of stay, days | 7 (4, 13) | 6 (3, 10) | <.001 ‡ |
| Hospital length of stay, days | 19 (10, 38) | 15 (9, 31) | .002 ‡ |
| ICU readmission | 59 (13%) | 68 (13%) | .9 † |
| Time of shock reversal, days, n=772 | 2.3 (1.5, 4.4) | 2.34(1.4, 4.2) | .6 ‡ |
| Incidence of new-onset arrhythmias | 198 (45%) | 221 (42%) | .4 † |
| AKI | 272 (62%) | 312 (60%) | .5 † |
| AKI stage |  |  | .4 † |
| I | 66 (24%) | 89 (29%) |  |
| II | 100 (37%) | 99 (32%) |  |
| III | 106 (39%) | 124 (40%) |  |
| Numbers indicate N (%) and (minimum, maximum) unless otherwise noted. † Chi-square ‡ Wilcoxon rank-sum | | | |
