# Supplementary material for: The order of vasopressor discontinuation and incidence of hypotension: a retrospective cohort analysis
Source: Sci Rep. 2021 Aug 17;11:16680. doi: 10.1038/s41598-021-96322-7 (PMC8371115; doi:10.1038/s41598-021-96322-7)
Supplement: Supplementary file 3 — Additional Table 3. Demographics and Patient Characteristics Among Hypovolemic Shock Only. [file 41598_2021_96322_MOESM3_ESM.docx]

**Additional Table 3.** Demographics and Patient Characteristics Among Hypovolemic Shock Only

| Characteristic | NE1 N=16 | VP1 N=16 | p-value |
| --- | --- | --- | --- |
| Age, yr | 71 (65, 76) | 72 (61, 79) | .9 † |
| Male sex | 10 (63%) | 9 (56%) | .7 ‡ |
| Weight, kg | 100 (84, 129) | 74 (60, 92) | .02 † |
| White race | 14 (88%) | 16 (100%) | .5 § |
| SOFA score | 9 (8, 10) | 9 (8, 12) | .7 † |
| Charlson comorbidity index | 7 (4, 7) | 5 (3, 7) | .5 † |
| Comorbid disease |  |  |  |
| Heart disease | 5 (31%) | 4 (25%) | 1.0 § |
| Pulmonary disease | 1 (6%) | 3 (19%) | .6 § |
| Immunodeficiency | 0 (0%) | 1 (6%) | 1.0 § |
| Liver disease | 0 (0%) | 1 (6%) | 1.0 § |
| Kidney disease | H | 1 (6%) | .08 § |
| Diabetes mellitus | 4 (25%) | 3 (19%) | 1.0 § |
| Cancer tumor | 3 (19%) | 4 (25%) | 1.0 § |
| Other | 3 (19%) | 4 (25%) | 1.0 § |
| Corticosteroid | 3 (19%) | 7 (44%) | .3 § |
| Requirement for dialysis | 2 (13%) | 2 (13%) | 1.0 § |
| Maximum NE dose; µg/kg/min | 0.10 (0.05, 0.15) | 0.20 (0.13, 0.28) | .002 † |
| Maximum VP dose; µg/kg/min | 0.04 (0.04, 0.04) | 0.04 (0.04, 0.04) | .3 † |
| NE end dose; µg/kg/min | 0.01 (0.01, 0.02) | 0.01 (0.01, 0.02) | .6 † |
| VP end dose; µg/kg/min | 0.02 (0.01, 0.04) | 0.02 (0.02, 0.04) | .5 † |
| VP within 3 hrs from shock start | 6 (38%) | 6 (38%) | 1.0 ‡ |
| MAP at first vasopressor initiation; mmHg | 69 (58, 80) | 72 (67, 79) | .5 † |
| MAP at first vasopressor discontinuation; mmHg | 73 (66, 81) | 79 (70, 81) | .4 † |
| Interventions after hypotension |  |  |  |
| Crystalloids >500ml | 16 (100%) | 16 (100%) |  |
| Albumin >25g | 16 (100%) | 16 (100%) |  |
| VP restart | 3 (19%) | 0 (0%) | .2 § |
| NE restart | 1 (6%) | 7 (44%) | .04 § |
| VP increase dose | 2 (13%) | 0 (0%) | .5 § |
| NE increase dose | 0 (0%) | 7 (44%) | .003 ‡ |
| Numbers indicate N (%) and (minimum, maximum) unless otherwise noted. † Wilcoxon rank-sum ‡ Chi-square § Fisher exact | | | |
