# Supplementary material for: The order of vasopressor discontinuation and incidence of hypotension: a retrospective cohort analysis
Source: Sci Rep. 2021 Aug 17;11:16680. doi: 10.1038/s41598-021-96322-7 (PMC8371115; doi:10.1038/s41598-021-96322-7)
Supplement: Supplementary file 4 — Additional Table 4. Clinical Outcomes of Hypovolemic Shock Only. [file 41598_2021_96322_MOESM4_ESM.docx]

**Additional Table 4.** Clinical Outcomes of Hypovolemic Shock Only

| Characteristic | NE1 N=16 | VP1 N=16 | p-value |
| --- | --- | --- | --- |
| Incidence of hypotension within 24 hours of first vasopressor stopped | 4 (25%) | 7 (44%) | .5 † |
| ICU mortality | 0 (0%) | 3 (19%) | .2 † |
| Hospital mortality | 2 (13%) | 3 (19%) | 1.0 † |
| 28-day mortality | 3 (19%) | 4 (25%) | 1.0 † |
| ICU length of stay, days | 5 (3, 5) | 6 (4, 8) | .3 ‡ |
| Hospital length of stay, days | 13 (9, 24) | 14 (8, 27) | .8 ‡ |
| ICU readmission | 1 (6%) | 2 (13%) | 1.0 † |
| Time of shock reversal, days, n=29 | 1.7 (1.0, 3.8) | 2.3 (1.6, 3.9) | .5 ‡ |
| Incidence of new-onset arrhythmias | 4 (25%) | 5 (31%) | 1.0 † |
| AKI | 6 (38%) | 9 (56%) | .3 § |
| AKI stage |  |  | .5 † |
| I | 1 (17%) | 2 (22%) |  |
| II | 1 (17%) | 4 (44%) |  |
| III | 4 (67%) | 3 (33%) |  |
| Numbers indicate N (%) and (minimum, maximum) unless otherwise noted. † Fisher exact ‡ Wilcoxon rank-sum § Chi-square | | | |
