# Supplementary material for: The order of vasopressor discontinuation and incidence of hypotension: a retrospective cohort analysis
Source: Sci Rep. 2021 Aug 17;11:16680. doi: 10.1038/s41598-021-96322-7 (PMC8371115; doi:10.1038/s41598-021-96322-7)
Supplement: Supplementary file 5 — Additional Table 5. Demographics and Patient Characteristics Among Cardiogenic Shock Only. [file 41598_2021_96322_MOESM5_ESM.docx]

**Additional Table 5.** Demographics and Patient Characteristics Among Cardiogenic Shock Only

| Characteristic | NE1 N=164 | VP1 N=93 | p-value |
| --- | --- | --- | --- |
| Age, yr | 64 (53, 74) | 66 (59, 74) | .1 † |
| Male sex | 116 (71%) | 71 (76%) | .3 ‡ |
| Weight, kg, n=250 | 87 (75, 101) | 86 (72, 100) | .8 † |
| White race | 145 (88%) | 87 (94%) | .4 § |
| SOFA score | 10 (8, 12) | 10 (9, 11) | .2 † |
| Charlson score | 5 (3, 7) | 5 (3, 7) | .5 † |
| Comorbid disease |  |  |  |
| Heart disease | 70 (43%) | 28 (30%) | .046 ‡ |
| Pulmonary disease | 22 (13%) | 10 (11%) | .5 ‡ |
| Immunodeficiency | 3 (2%) | 5 (5%) | .1 § |
| Liver disease | 3 (2%) | 5 (5%) | .1 § |
| Kidney disease | 48 (29%) | 23 (25%) | .4 ‡ |
| Diabetes mellitus | 37 (23%) | 32 (34%) | .04 ‡ |
| Cancer tumor | 34 (21%) | 16 (17%) | .5 ‡ |
| Other | 18 (11%) | 23 (25%) | .004 ‡ |
| Corticosteroid | 35 (21%) | 20 (22%) | .9 ‡ |
| Requirement for dialysis | 25 (15%) | 13 (14%) | .8 ‡ |
| Maximum NE dose; µg/kg/min | 0.10 (0.06, 0.15) | 0.12 (0.09, 0.20) | .005 † |
| Maximum VP dose; µg/kg/min | 0.04 (0.04, 0.04) | 0.04 (0.04, 0.04) | .02 † |
| NE end dose; µg/kg/min; n=256 | 0.01 (0.01, 0.02) | 0.01 (0.01, 0.02) | .1 † |
| VP end dose; µg/kg/min | 0.02 (0.01, 0.04) | 0.02 (0.01, 0.03) | .3 † |
| VP within 3 hrs from shock start | 75 (46%) | 48 (52%) | .4 ‡ |
| MAP at first vasopressor initiation; mmHg | 71 (64, 82) | 69 (62, 77) | .1 † |
| MAP at first vasopressor discontinuation; mmHg | 79 (71, 88) | 78 (68, 88) | .6 † |
| Interventions after hypotension |  |  |  |
| Crystalloids >500ml | 1 (1%) | 0 (0%) | 1.0 § |
| Albumin >25g | 1 (1%) | 0 (0%) | 1.0 § |
| VP restart | 18 (11%) | 0 (0%) | <.001 ‡ |
| NE restart | 1 (1%) | 22 (24%) | <.001 § |
| VP increase dose | 12 (7%) | 0 (0%) | .008 ‡ |
| NE increase dose | 1 (1%) | 17 (18%) | <.001 § |
| Numbers indicate N (%) and (minimum, maximum) unless otherwise noted. † Wilcoxon rank-sum ‡ Chi-square § Fisher exact | | | |
