# Supplementary material for: The order of vasopressor discontinuation and incidence of hypotension: a retrospective cohort analysis
Source: Sci Rep. 2021 Aug 17;11:16680. doi: 10.1038/s41598-021-96322-7 (PMC8371115; doi:10.1038/s41598-021-96322-7)
Supplement: Supplementary file 6 — Additional Table 6. Clinical Outcomes of Cardiogenic Shock Only. [file 41598_2021_96322_MOESM6_ESM.docx]

**Additional Table 6.** Clinical Outcomes of Cardiogenic Shock Only

| Characteristic | NE1 N=164 | VP1 N=93 | p-value |
| --- | --- | --- | --- |
| Incidence of hypotension within 24 hours of first vasopressor stopped | 21 (13%) | 22 (24%) | .03 † |
| ICU mortality | 24 (15%) | 20 (22%) | .2 † |
| Hospital mortality | 27 (17%) | 23 (25%) | .1 † |
| 28-day mortality | 32 (20%) | 25 (27%) | .2 † |
| ICU length of stay, days | 6 (3, 12) | 6 (4, 11) | .3 ‡ |
| Hospital length of stay, days | 17 (9, 27) | 19 (11, 27) | .5 ‡ |
| ICU readmission | 19 (12%) | 18 (19%) | .1 † |
| Time of shock reversal, days, n=213 | 2.4 (1.6, 6.0) | 3.3 (1.6, 5.3) | .4 ‡ |
| Incidence of new-onset arrhythmias | 85 (52%) | 50 (54%) | .8 † |
| AKI | 98 (60%) | 55 (59%) | .9 † |
| AKI stage |  |  | .6 † |
| Missing | 66 | 38 |  |
| I | 34 (35%) | 20 (36%) |  |
| II | 30 (31%) | 20 (36%) |  |
| III | 34 (35%) | 15 (27%) |  |
| Numbers indicate N (%) and (minimum, maximum) unless otherwise noted. † Chi-square ‡ Wilcoxon rank-sum | | | |
