# Supplementary material for: The order of vasopressor discontinuation and incidence of hypotension: a retrospective cohort analysis
Source: Sci Rep. 2021 Aug 17;11:16680. doi: 10.1038/s41598-021-96322-7 (PMC8371115; doi:10.1038/s41598-021-96322-7)
Supplement: Supplementary file 7 — Additional Figure 1. Cumulative incidence of hypotension following first vasopressor discontinuation among septic shock only patients. [file 41598_2021_96322_MOESM7_ESM.docx]

**
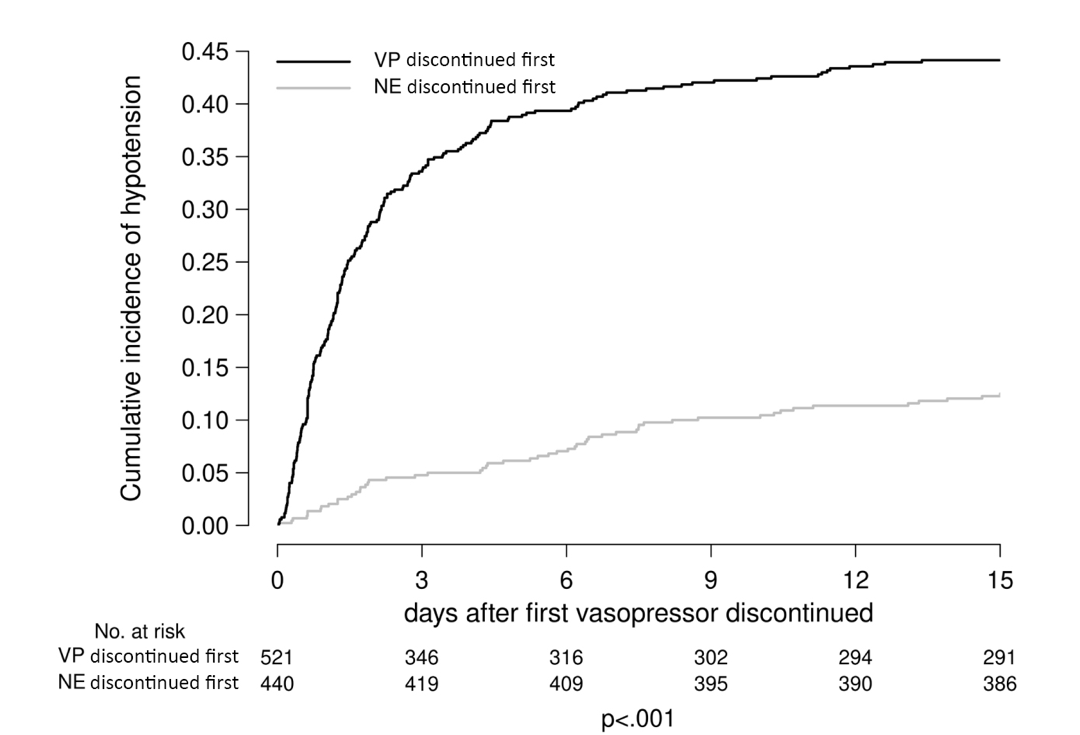
**

**Additional Figure 1. Cumulative incidence of hypotension following first vasopressor discontinuation among septic shock only patients.**
